# Benefits of Treadmill Training for Patients with Down Syndrome: A Systematic Review

**DOI:** 10.3390/brainsci13050808

**Published:** 2023-05-16

**Authors:** Karolina Kamińska, Michał Ciołek, Krzysztof Krysta, Marek Krzystanek

**Affiliations:** 1 Students’ Scientific Association, Department and Clinic of Rehabilitation Psychiatry, Faculty of Medical Sciences in Katowice, Medical University of Silesia in Katowice, 40-055 Katowice, Poland; karolina.kaminska311@gmail.com (K.K.); m.cioleksl@gmail.com (M.C.); 2 Department and Clinic of Rehabilitation Psychiatry, Faculty of Medical Sciences in Katowice, Medical University of Silesia in Katowice, 40-055 Katowice, Poland; krzystanekmarek@gmail.com

**Keywords:** Down syndrome, treadmill training, physiotherapy, intellectual disability

## Abstract

Background: The objective of this study was to evaluate the effectiveness of various results of treadmill training in children and adults with Down syndrome (DS). Methods: To provide an overview of this effectiveness, we conducted a systematic literature review of studies in which participants with DS from all age groups received treadmill training, alone or combined with physiotherapy. We also looked for comparisons with control groups of patients with DS who did not undergo treadmill training. The search was performed in medical databases: PubMed, PEDro, Science Direct, Scopus, and Web of Science, and included trials published until February 2023. Following PRISMA criteria, the risk of bias assessment was conducted using a tool developed by the Cochrane Collaboration for RCT. The selected studies presented multiple outcomes with differences in methodology; therefore, we were not able to conduct any sort of data synthesis, so we present measures of treatment effect as mean differences and corresponding 95% confidence intervals. Results: We selected 25 studies for the analysis with a total number of 687 participants, and identified 25 different outcomes which are presented in a narrative manner. In all outcomes we observed positive results favoring the treadmill training. Discussion: Introducing treadmill exercise into typical physiotherapy generates improvement in mental and physical health of people with DS.

## 1. Introduction

Down syndrome (DS) is a genetic disorder that affects people of all races and societies, occurring in approximately 1 in every 1000–1100 children. It is caused by either partial or complete triplication of chromosome 21, and is considered to be one of the most prevalent causes of intellectual disability globally [1]. There are three genetic types of Down syndrome: trisomy 21, mosaicism, and Robertsonian translocation [2].

### 1.1. Health Complications Associated with Down Syndrome

It has been reported that people with Down syndrome struggle with many health problems and their life expectancy (approximately 55 years) is shorter than in neurotypical individuals. Furthermore, its incidence has been increasing over the years [3]. The reported health problems include congenital heart defects, hypothyroidism, leukemia, coeliac disease, muscle hypotonia, ligamentous laxity, atlantoaxial instability, epilepsy, obstructive sleep apnea, autoimmune diseases, recurrent respiratory infections, hearing and vision problems, early onset Alzheimer disease, and anxiety disorder [3,4]. 

### 1.2. Motor Development and Cognitive Function in Down Syndrome

Low muscle tone and ligamentous laxity results in delay of walking onset in infants with Down syndrome. They stand alone and walk independently when they are 9, 18, or 19 months old, whereas neurotypical infants usually walk when they are 6, 11, or 12 months old [5,6]. The atypical motor behavior of infants with Down syndrome is seen even in the first weeks of their life and leads to various implications for their future motor skills, for example, weaker grip strength and difficulty maintaining body posture [7,8]. Independent walking is also important because of its influence on cognitive and social development [9]. 

### 1.3. Obesity and Alzheimer’s Disease in Down Syndrome

People with Down syndrome also have a higher risk of obesity, which is associated with a pro-inflammatory status [10]. Furthermore, they are susceptible to early progressive loss in executive functions (cognitive strategies to process adaptive, goal-directed actions) which is associated with early onset Alzheimer’s disease in individuals with Down syndrome. Postmortem analyses showed the presence of beta-amyloid plaques, characteristic features of the development of Alzheimer’s disease, in almost all of the neurological tissue of people with Down syndrome aged 35–40 years [11]. These plaques were also found in 8-year-old children [12].

### 1.4. Treadmill Training as a Physical Therapy Method

Studies have shown that adolescents who have Down syndrome exhibit weaker working memory, slower processing speed, and poor attention span and inhibitory control when compared to their peers who are typically developing. It surely degrades their quality of life [13,14]. Individuals with Down syndrome also have difficulty in productivity of words and switching in the semantic and the phonological fluency test compared with neurotypical persons [15]. It is widely accepted that treadmill training is a universal physical therapy method for supporting the treatment of many various conditions, for example, Parkinson’s disease [16], chronic stroke [17], and cerebral palsy [18]. Physical therapy incorporates treadmill training as a means to enhance the locomotor functions of patients, which is made feasible by the plasticity of the central nervous system. The activation of trophic factors, neurogenesis, synaptogenesis, and angiogenesis during treadmill training leads to this improvement, especially in the early stages of development [19]. According to reports, a single 30 min exercise session could enhance the information processing speed and executive function of teenagers and young adults who have Down syndrome [20].

### 1.5. Evaluating the Effectiveness of Treadmill Training for Down Syndrome

It might be worthy of attention to evaluate the effectiveness of treadmill training, not only to improve locomotor performance, but also more universally, in various problems that people with Down syndrome struggle with, especially those related to the brain functions (executive and cognitive) or their inflammatory system. There are several existing systematic reviews that focus only on one of these functions or not particularly on treadmill training, or not only in patients with Down syndrome, and often describe only studies related to children [21,22,23,24,25,26,27]. The aim of this review is to address the existing gap in accessible analyses and evaluate the effectiveness of various results of treadmill training in children and adults with Down syndrome, because up until the present time there are no available systematic reviews on this topic.

## 2. Materials and Methods

This systematic review was conducted according to reporting standards of the PRISMA protocol [28]. The review was registered at the International Prospective Register of Systematic Reviews PROSPERO with the following number: CRD42023412948.

### 2.1. Research Question

The research question was formulated using the PICO format. We aimed to analyze the benefits of treadmill training alone or combined with physiotherapy in study groups compared to control groups, in which no interventions were applied, in patients with Down syndrome.

### 2.2. Objectives of Systematic Review

The objectives of this systematic review were to:

- Determine various effects of treadmill training, alone or combined with physiotherapy among children and adults with Down syndrome;
- Assess the quality of the included RCTs using the Cochrane risk-of-bias tool for randomized trials;
- Analyze and compare the results of the selected studies.

### 2.3. Search Strategy and Selection Process

**Figure 1:**
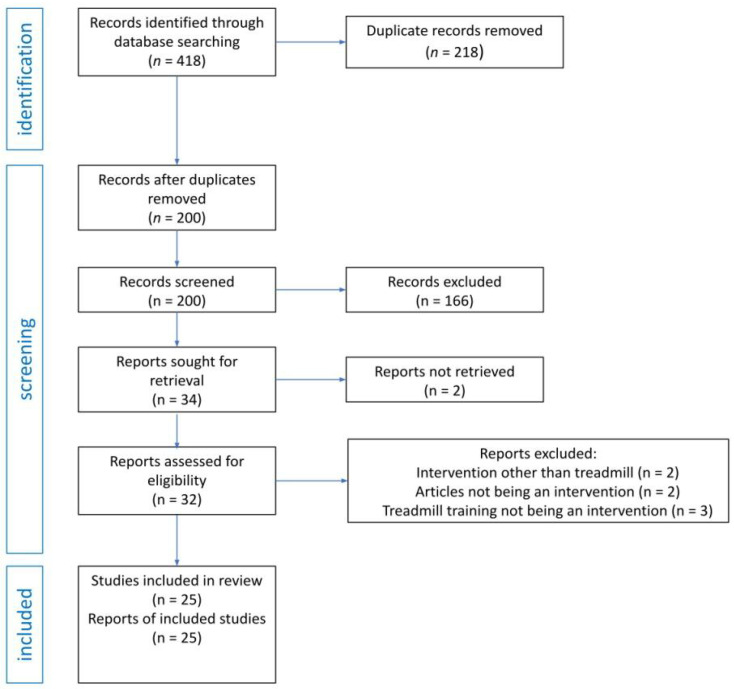
The search process.

The search was conducted on 21 February 2023 in five online medical databases: PubMed, PEDro, Science Direct, Scopus, and Web of Science. All databases were searched using the same search query: (“Down Syndrome”) AND (“treadmill”) in the title, abstract, and keywords fields. These search terms were developed based on preliminary searches carried out in each selected database. All identified records were documented and screened in Mendeley reference manager, which was also used for duplicate removal. Both authors independently screened titles and abstracts against the eligibility criteria of the review. Cases of disagreement were discussed until a consensus was reached. The search process is illustrated in Figure 1.

### 2.4. Eligibility Criteria

- Types of studies: Randomized controlled, quasi-experimental, or clinical trial or pilot study published in English or Polish in peer-reviewed journals published from the inception of the database until 21st February 2023.
- Participants: Study participants with Down syndrome from all age groups.
- Intervention: Studies in which participants undergo treadmill training, alone or combined with physiotherapy.
- Comparison: Control groups formed only with patients with DS, who were offered only standard physiotherapy care or no therapeutic intervention.
- Outcome: In order to be included in the analysis, the study had to use a defined clinical outcome relating to mental or physical health in Down syndrome.

### 2.5. Risk of Bias Assessment

Following the Preferred Reporting Items for Systematic Reviews and Meta-Analyses (PRISMA) criteria [28], the risk of bias (RoB) assessment was conducted using a tool developed by the Cochrane Collaboration for randomized controlled trials [29]. 

### 2.6. Data Extraction

Data were extracted from each of the included reports using the Data Collection Form for Intervention Reviews for RCTs and non-RCTs template, consistent with the Cochrane Handbook for Systematic Reviews of Interventions [30].

### 2.7. Data Analysis

In the current review, we aim to summarize and analyze various types of outcome because in the selected articles we identified differences in study designs, effect measures, and clinical questions. Moreover, the age spectrum of the participants was wide, from infants to elderly people. Taking into consideration all the reasons presented above, we report all the data in a narrative review instead of in a meta-analysis. All outcome measures in this review are continuous. In order to measure the treatment effect, we used the mean difference and corresponding 95% confidence intervals.

## 3. Results

A total of 418 articles we originally identified. Within those results, duplicate removal was conducted using Mendeley reference manager and manual searching, which resulted in 200 records. Next, 166 articles were excluded based on title and abstract evaluation, mostly because the article was not an intervention study (specifically, reviews, book reviews, media reviews, editorials, obituaries, and case studies), or it did not have an eligible design, it recruited participants without DS, or a treadmill was not used as an intervention. Two articles were excluded because they could not be obtained. Therefore, 32 reports underwent full-text examination, after which 7 were excluded: 2 due to an intervention other than treadmill, 2 due to not being an intervention, and 3 due to treadmill training not being an intervention. Finally, 25 articles met the inclusion criteria. 

The results of the search are presented in Table 1.

### 3.1. Study Characteristics of Included Studies

We included 25 studies with a total number of 687 participants [8,31,32,33,34,35,36,37,38,39,40,41,42,43,44,45,46,47,48,49,50,51,52,53,54]. The real number of subjects included in this analysis might be different because seven of the trials were performed with the same experimental group [36,37,38,39,40]. Papers by Ulrich et al. (1992) [31] and Ulrich et al. (1995) [32] had the same method of recruitment and intervention and the same number of participants. Similar situations happened in the case of trials by Ordonez et al. (2013) [46], Ordonez et al. (2014) [48], and Rosety-Rodriguez et al. (2014) [51]. Six studies [50,52,53,54] had the same method of recruitment and the same type of intervention. In the manuscripts by Wu et al. (2007) [33] and Ulrich et al. (2001) [36], there was the same control group.

#### 3.1.1. Study Design

Nine studies were randomized controlled trials [8,33,36,42,43,44,50,52], six trials used a randomized parallel group design with no control group [37,38,39,40,44,47], six were quasi-experimental designs [34,35,44,51,53,54], and four were single-group designs [31,32,41,49].

#### 3.1.2. Setting 

Fourteen studies were conducted in the USA [31,32,33,36,37,38,39,40,42,43,50,52,53,54], four in Spain [44,47,48,51], two in Israel [34,35], two in Egypt [8,45], one in Portugal [41], one in Taiwan [46], and one in Brazil [49].

In eleven studies, interventions were provided at participants’ homes [31,32,33,36,37,38,39,40,42,43]. In the study by Looper et al. (2010) [42], interventions were also located at the motor development laboratory. In the El-Meniawy et al. (2011) trial [45], the intervention took place in the outpatient clinic of The Faculty of Physical Therapy in Cairo University. Lin et al. (2012) [47] performed their study in a 48 m² room located at the Department of Occupational Therapy of Kaohsiung Medical University. Other studies did not include information about their settings.

#### 3.1.3. Participants

##### Characteristics

All studies [8,31,32,33,34,35,36,37,38,39,40,41,42,43,44,45,46,47,48,49,50,51,52,53,54] focused on people diagnosed with Down syndrome, both in the experimental and control groups. The most common comorbidities were those associated with the cardiovascular system, for example, congenital heart defects [31,32,33,38], cardiac disease [34], arterial occlusive disease [35], and obesity [46,48,51]. The percentage of male participants was approximately 53.86%. In the studies of Ulrich et al. (2001) [36], Looper et al. (2010) [42], and Chen et al. (2016) [53], there was no information about this. In Alskhawi et al. (2019) [8] and El-Meniawy et al. (2011) [45], there was information indicating that both sexes participated in the study. The trials of Mendonca et al. (2009) [41], Ordonez et al. (2010) [44], and Chen et al. (2014) [50] included only males, while those of Ordonez et al. (2013) [46], Ordonez et al. (2014) [48], and Rosety-Rodriguez et al. (2014) [51] included only females. The age of patients varied substantially. Some of the studies referred to infants [31,32,33,36,37,38,39,40,42,43] from 8 months [32] to 75 months [43], and children and adolescents [44,45,47,49,54] from 4.5 years [8] to 16.3 years old [44]. Other studies [34,35,41,46,48,50,51,52,53,54] involved adults with Down syndrome, with a mean age of 24. The information about race or ethnicity was only known in 5 studies: Ulrich et al. (2001) [33] (2 mixed race and 28 white); Ulrich et al. (2007) [38] (2 African American, 26 white, and 2 biracial); Angulo-Barroso et al. (2008) [39] (2 African American, 26 Caucasian, and 2 other); Mendonca et al. (2009) [41].

##### Number of Participants, Method of Recruitment, and Other Relevant Information

Ulrich et al. (1992) [31] enrolled seven infants, all of them to the experimental group. They were recruited from the Down Syndrome Support Association of Central Indiana. These infants received physical therapy from 30 min to 1 h per week before the beginning of the study. Ulrich et al. (1995) [32] enrolled seven infants, all in the experimental group. They were recruited from the same place as in the Ulrich 1992 trial. Two infants were born prematurely. Ulrich et al. (2001) [33] conducted another study, which involved recruiting a total of 30 participants from both parent support groups and DS clinics, with 15 participants in each of the experimental and control groups. Before the experiment, they received traditional physical therapy every week. Carmeli et al. (2002) [34] enlisted 26 inhabitants of an Israeli foster home, with 16 assigned to the experimental group and 10 assigned to the control group. It was ensured that the subjects did not consume any drugs that could have impeded their balance or strength performance. Additionally, Carmeli et al. (2004) [35] conducted another study with 26 residents of an Israeli foster home, in which 14 individuals were placed in the experimental group and 12 were placed in the control group. Six participants in the experimental group had symptoms of intermittent claudication. The paper by Wu et al. (2007) [36] involved the enrollment of 45 participants, with 30 assigned to the experimental group and 15 to the control group. The study included two cohorts of participants from two separate studies. The initial cohort was comprised of infants with DS who were recruited when they were capable of completing six steps per minute while receiving assistance from a treadmill. Participants were sourced from parent support groups in Lower Michigan. The control group for the analysis was taken from another study, by Ulrich et al. (2001) [33], who enrolled patients from parent support groups and DS clinics when they could sit alone for 30 s. Studies by Wu et al. (2008) [37], Ulrich et al. (2007) [38], Angulo-Barroso et al. (2008) [39], Angulo-Barroso, Wu et al. (2008) [40], and Wu et al. (2010) [43] each involved 30 participants, who were members only of the experimental groups, which were the same groups as in the paper by Wu et al. (2007) [36]. Mendonca et al. (2009) [41] enrolled 12 participants, all of them to the study group. They were recruited from a vocational center dedicated to the professional employability of individuals with intellectual disabilities. At the entry to the study, the subjects’ average weight was 66.9 ± 8.9 kg and their height was 155.4 ± 9.5. In the study conducted by Looper et al. (2010) [42], a total of 17 participants with DS were included, with 10 assigned to the experimental group and 7 to the control group. The study by Ordonez et al. (2010) [44] included 38 participants (31 in the experimental group and 7 in the control group). Thirty participants took part in the study by El-Meniawy et al. (2012) [45]; all of them were members of the experimental group, but they were divided into two equal groups. In the work by Lin et al. (2012) [47], 92 individuals with disabilities from the Kaohsiung and Pingtung metropolitan areas met the inclusion criteria for the study (46 of them were in each of the experimental and control groups). School nurses, teachers, and directors selected individuals eligible for the experiment. In the study conducted by Ordonez et al. (2013) [46], they selected 20 women with Down syndrome who were obese, of which 11 were in the experimental group and 9 were in the control group. The participants did not have any harmful habits such as smoking or alcohol consumption, and were not taking any medication that could affect their appetite regulation or physical performance. To ensure that diet did not affect the results, the parents of the participants were carefully instructed to avoid any differences in the quantity or quality of food given to the control and experimental groups. In a similar study by Ordonez et al. (2014) [48], 20 obese women with DS were also enrolled (11 to the experimental group and 9 to the control group). They were recruited from different community groups for people with ID and their families. In the experiment by Rodenbush et al. (2013) [49], the participants were 16 non-probabilistically chosen subjects from 2 rehabilitation centers (the Association of Parents and Friends of Exceptional Children—APAE—and the Association to Assist Disabled People—ADOTE). There was no control group. Rosety-Rodriguez et al. (2014) [51] had the same experimental and study group as that of Ordonez et al. (2014) [48]. Chen et al. (2014) [50] recruited 20 young men—12 to the experimental group and 8 to the control group. In the studies by Chen et al. (2015 [52], 2016 [53], and 2019 [54]) the investigators invited participants recruited from local Special Olympic programs or DS organizations (e.g., Sharing Down syndrome Arizona, DS Network Arizona). Chen et al. (2015) [52] enrolled 20 young adults (10 in each of the experimental and study groups). In the study by Chen et al. (2016) [53] there were 18 young adults (12 in the experimental group and 6 in control group). Chen et al. (2019) [54] recruited 28 young adults (18 to the experimental group and 10 to the control group). Alsakhawi et al. (2019) [8] invited 45 participants recruited from an outpatient clinic at the Faculty of Physical Therapy in Cairo University. There were three equal groups: A, B and C. The inclusion criteria in studies focusing on infants referred to the ability to take a minimum of six spontaneous steps on a treadmill [37,38,39,40,43]. The exclusion criteria often refer to pre-existing conditions that may affect the ability to undergo the intervention, for example blindness, seizure disorder, musculoskeletal and auditory problems [34,36,38,40,41,42,45,46], mental age of participants lower than 3 years [50,52,53,54], or hypothyroidism [48]. Following the application of these criteria and occurrence of other conditions, there were some cases of withdrawing the participants from the studies described in the above table [32,36,38,39,42,43,46,49]. The information about ethical approval of the studies was included in all of them except for two [31,45]. The information about informed consent obtained from the participants was included in all the studies except for one [31].

#### 3.1.4. Interventions

##### Treadmill Training Interventions in Various Studies

Treadmill training was the form of intervention applied in all of the twenty-six studies [8,31,32,33,34,35,36,37,38,39,40,41,42,43,44,45,46,47,48,49,50,51,52,53,54]. Specifications such as speed, duration, and inclination of treadmill exercise were adapted to the aims of the studies and the characteristics of the participants. In the study by Ulrich et al. (1992) [31], the treadmill training was performed 8 times for 30 s, and the speed of this training was 0.1 m/s, 0.15 m/s, and 0.2 m/s, in each set in 6 of 8 trials. Infants were supported by the investigators under the arms. The same method of intervention was used by Ulrich et al. (1995) [32], but additional information was added that the infants perform training trials monthly for several months, until every participant demonstrated consistent alternating step patterns across three successive testing sessions. Ulrich et al. (2001) [33] conducted an experiment in which the treadmill training was conducted for eight minutes a day, five days a week, until the participants displayed the capability to walk independently. The speed of the training was set at 2 m/s, and the infants were supported by their parents. Infants began participating in the study when they were able to sit independently for 30 s. The study was monitored by a team of researchers who visited all participants biweekly. Carmeli et al. (2002) [34] and Carmeli et al. (2004) [35] instructed the participants to walk at a comfortable pace, and if required, they could hold onto the handrails for balance adjustments while walking. Prior to the training, a warm-up session was conducted for 3 min, followed by knee extension and flexion exercises in the sitting position, and then 5 min of active stretching. The participants only walked between 9:30 a.m. and 11:30 a.m. indoors, and the environmental conditions were controlled to maintain a temperature of 23 °C and 40% humidity. In the study by Carmeli et al. (2002) [34], treadmill training was performed 3 times per week for 25 consecutive weeks, initially for 10–25 min, then increasing to 45 min. In the experiment of Carmeli et al. (2004) [35], treadmill training was performed 3 times per week, for 15 consecutive weeks, initially 5–15 min, then increasing to 40 min. The control groups did not undergo treadmill exercises. The study by Wu et al. (2007) [36] had an experimental group which consisted of two subgroups. The first was a lower-intensity-generalized (LG) training group, which included 14 participants, while the second subgroup was a higher-intensity-individualized (HI) training group, which was composed of 16 participants. The same groups of participants can be found in the publications by Wu et al. (2008) [37], Ulrich et al. (2007) [38], Angulo-Barroso et al. (2008) [39], Angulo-Barroso and Wu et al. (2008) [40], and Wu et al. (2010) [43]. In studies by Wu et al. (2007) [36], Wu et al. (2008) [37], Angulo-Barroso and Wu et al. (2008) [40], and Wu et al. (2010) [43], the LG group performed treadmill training for 6 min per day for 5 days a week; the HI group trained for 5 days per week, but the participants had individualized programs. The speed was 0.18 m/s. In works by Ulrich et al. (2007) [38] and Angulo-Barroso et al. (2008) [39], the LG group performed treadmill training for 8 min per day, 5 days per week; the HI group trained for 8–12 min a day. The speed for the LG group was 0.15 m/s and that for the HI group 0.15–0.3 m/s. Infants were supported by their parents in all studies [36,37,38,39,40,43]. Throughout the pre-walking phase of the study by Wu et al. (2007) [36], the research team made biweekly visits to all participants in order to measure their physical dimensions, record a 5 min period of treadmill stepping, and ensure that the training program was being followed as necessary. In a subsequent gait follow-up, participants were asked to walk at their own pace on the GAITRite mat, with measurements taken from an average of four walking trials. Participants in the control group did not engage in the treadmill training program but were instead asked to walk at their own pace across an 8-foot walkway that was covered with a long strip of 3-foot-wide butcher paper during the follow-up. The research performed by Wu et al. (2008) [37] also involved regular visits to the infants’ homes by the staff during the treadmill intervention. The high-intensity (HI) group utilized ankle weights ranging from 0 to 115% of their calf mass. Thirteen individuals from each group completed a one-year follow-up of their gait after the treadmill intervention. The initial visit was scheduled as soon as parents reported that their child could walk 8–10 steps continuously at home, which took approximately 3 months for both groups. Ulrich et al. (2007) [38] conducted an experiment on the HI group where ankle weights were added, equivalent to 125% of the calf mass circumference, along with increased belt speed and daily duration to optimize the stepping response. The researchers visited all families every two weeks to ensure that the infants were following the treadmill training protocols. They also recorded five 1 min trials of the infants stepping while being supported on the treadmill, measured body weight, height, and shank length, and answered questions from the caregivers. In the work by Angulo-Barroso et al. (2008) [39], the HI group participated in the treadmill intervention, with progressively increasing treadmill belt speed, time, and ankle weights (to 125% of calf mass). In the study by Angulo-Barroso and Wu et al. (2008) [40], the research staff visited all the participants biweekly throughout the treadmill intervention. The HI group had an ankle weight that was 14–115% of calf mass. There was a one-year gait follow-up after the treadmill intervention—thirteen new walkers in the LG group (four male, nine female) and twelve in the HI group (nine male, three female) came into the laboratory immediately after walking onset. In the paper by Wu et al. (2010) [43], as soon as the parents informed the researchers that their child could take 8 to 10 independent steps at home, the initial visit was promptly arranged. The papers authored by Wu et al. (2007) [36], Angulo-Barroso et al. (2008) [39], Angulo-Barroso and Wu et al. (2008) [40], and Wu et al. (2010) [43] all had a similar scheduling pattern for the second, third, and fourth gait visits, which were set to occur three, six, and twelve months, respectively, after the first visit. In the experiment by Mendonca et al. (2009) [41], treadmill training was performed 8 min (submaximal protocol) and 12 min (graded maximal exercise protocol) 2 times per week for 28 weeks. The submaximal protocol was at 2.5 km/h and there was a 0% grade. The graded maximal exercise protocol was at 4 km/h and increased by 1.6 km/h every minute until exhaustion, and the grade increased by 2.5% every 2 min until 12.5% was reached. Before the study, participants received pre-training for 2 times a week for 12 weeks, as follows: 30 min (treadmill), 5 min warm-up dynamic activities, 40 min of continuous ergometer conditioning (treadmill, stepper, upright stationary cycle, and rowing ergometer), followed by a 5 min dynamic cool-down.

##### Treadmill Training with Orthoses and Virtual Reality

In Looper and colleagues’ study (2010) [42], children wore orthoses throughout the entire study, which involved training sessions lasting 8 min per day, 5 days per week. The training continued until the children could take three independent steps, and the infants were assisted by their parents during the process. Between walking onset and follow-up testing, the children underwent treadmill training at a speed of 0.2 m/s while wearing the orthoses. After one month from the walking onset, the developmental tests were administered again. The control group did not wear orthoses. In the study by Ordonez et al. (2010) [44], there was a 12-week treadmill training program (3 days per week for 20–35 min, increasing by 5 min each 3 weeks). The participants also received a warm-up (15 min) and cool-down (10 min). The work intensity on the treadmill was 60–75% of peak heart rate. The control group did not undergo treadmill training. In the study by El-Meniawy et al. (2012) [45], the duration of treadmill training was 20 min, 3 times a week for 3 months with 75% of over-ground speed. There was a warm-up for 5 min and cool-down for 5 min. Each group received an exercise program for balance and posture control for 30 min. Data were also collected three months after the treatment. In the experiment by Lin et al. (2012) [47], treadmill training took place for 5 min, 3 times a week for 6 weeks. The speed varied between 2.0 (0% incline) and 3.0 kph (5% elevation). Additionally, there was a warm-up (10 min), and a single virtual reality-based activity lasted for 20 min, administered 3 times per week for a duration of 6 weeks, with a 10 min break. There was a follow-up after 6 weeks. The control group did not undergo treadmill training.

##### Treadmill Training Duration and Intensity

Ordonez et al. (2013) [46], Ordonez et al. (2014) [48], and Rosety-Rodriguez et al. (2014) [51] conducted studies in which treadmill training lasted for 30–40 min, 3 times per week, over a period of 10 weeks. The training duration was gradually increased by 2.5 min every 2 weeks. The participants began with a 10–15 min warm-up period and ended with a 5–10 min cool-down period. The initial stage involved walking at a speed of 4.0 km/h for 2 min. The incline of the treadmill was then raised by 2.5% every 2 min until it reached a grade of 12.5%. The grade was then kept constant while the speed was increased by 1.6 km/hr every minute until the point of exhaustion. The work intensity on the treadmill was 55–65% of peak heart rate. Rosety-Rodriguez et al. (2014) [51] conducted re-evaluations of the parameters at 1, 3, and 6 months following the completion of the training program, during which time the participants did not engage in any further training program. In the experiment by Rodenbusch et al. (2013) [49], the treadmill training lasted 2 min for each inclination (0–10%). Speed was comfortable for participants. There was a 1 min rest period between changes in inclination. Data were captured for 30 s in each inclination.

Chen et al. (2014) [50], (2015) [52], (2016) [53], and (2019) [54] conducted studies where the training session was 20 min long, with a speed of 2.0–3.0 mph. The treadmill incline was increased by 2.5% every 4 min until the walking protocol was completed, which involved 5 stages and a 0–10% incline. Prior to the training, a warm-up was performed, where the speed was increased from 0.5 mph to 2.0 mph, with increments of 0.5 mph per minute. The walking protocol was stopped if the participant’s heart rate exceeded 85% of their predicted maximum, the entire protocol was completed, or the participant reported feeling too tired or being unwilling to continue. In the work by Chen et al. (2015) [52], during the last 15 s of each stage, the participants were asked to select a picture on the rating of perceived exertion (RPE) scale in response to their perception of exertion. In the experiment by Chen et al. (2016) [54], the participants were assigned to high-intensity exercise (i.e., 75–85% of predicted maximum heart rate) (N = 6) or moderate-intensity exercise (i.e., 50–75% of predicted maximum heart rate) (N = 6). Chen et al. (2019) [55] conducted a study with two groups of participants who performed either high- or moderate-intensity exercise, with the former involving heart rates of 70–85% of predicted maximum and the latter involving heart rates of 50–69% of predicted maximum. After the intervention, participants were given 5–10 min of rest before taking the verbal fluency test again as a post-test measure. The total duration of the testing period was about an hour and a half. Control groups in all studies spent 20 min watching a video.

##### Combining Treadmill Training with Other Physical Therapy Interventions

Alsakhawi et al. (2019) [8] conducted a study with three groups. Group A received traditional physical therapy for 60 min to improve balance in the participating children. Group B underwent the same therapy as Group A for 30 min and additional core stability exercise training. Group C followed the same intervention strategies as Group A for 30 min, combined with a treadmill exercise program (20 min, three times a week, for eight weeks at 75% of over-ground speed and individually prescribed low-endurance walking). Before each walking session, the children in all groups engaged in 5 min of active stretching exercises that involved prolonged and progressive stretching of the hamstrings, quadriceps muscles, and Achilles tendon, followed by 30 min of physical therapy.

#### 3.1.5. Outcome Assessment Tools

##### Walking Onset and Gait Patterns in Infants

The Bayley Scales of Infant Development was used in the studies by Ulrich et al. (1995) [32], Ulrich et al. (2001) [33], Ulrich et al. (2007) [38], Angulo-Barroso et al. (2008) [39], and Angulo-Barroso and Wu (2008) [40]. To measure the motor skills (four step types—alternating, single, parallel, and double) in Ulrich et al. (1992) [31] and Ulrich et al. (1995) [32], researchers used the camera. The GAITRite system and the camera were used in studies by Wu et al. (2007) [36], Wu et al. (2008) [37], and Angulo-Barroso et al. (2008) [39] to measure average velocity, stride length, step width, stride time, stance time, and dynamic base of infants. An ankle band with an activity monitor was used in the study by Angulo-Barroso and Wu et al. (2008) [40]. In the experiment by Wu et al. (2010) [43], the researchers used markers to measure the kinematics of the hip, knee, and ankle joints. The study conducted by Looper et al. (2010) [42] utilized Gross Motor Function as a tool for assessing gross motor skill development in various positions and movements, including lying and rolling, sitting, crawling and kneeling, and standing, as well as walking, running, and jumping.

##### Motor and Cardiovascular Function

In the study by Carmeli et al. (2002) [34], a dynamometer and the “timed get-up and go” test were applied to measure dynamic balance. In the study by Carmeli et al. (2004) [35], researchers used Vasculab PPG, Pain Physiopathology Index (PPI), and ABI to assess the volume of blood present in capillaries and pain levels. In the experiment by Mendonca et al. (2009) [41], a standardized body composition assessment was used (anthropometric measurements and bioelectrical impedance spectroscopy), and the following tools were applied: a resting protocol, submaximal steady-state exercise protocol, and a maximal graded exercise protocol to measure VO2 and pulmonary minute ventilation elicited by the selected submaximal treadmill exercise task and peak VO2. A Biodex instrument system was applied to assess stability level, feet angles, and heel coordinates in the study by El-Meniawy et al. (2011) [45]. In the experiment by Lin et al. (2012) [47], a handheld dynamometer, the Bruininks–Oseretsky Test of Motor Proficiency—Second Edition, a study questionnaire, and the Wechsler Intelligence Scale for Children—Third Edition were used to measure muscle strength (hip extensor, hip flexor, knee extensor, knee flexors, hip abductors, and ankle plantarflexor) and agility performance. In the work by Rodenbusch et al. (2013) [49], GMFCS, Berg Balance Scale (BBS), and the Qualisys Motion Capture System helped to evaluate spatial-temporal variables and angular variation of the hip, knee, and ankle in the sagittal plane. A hydraulic dynamometer was used in the study by Chen et al. (2014) [50] to measure grip strength. In the study by Alsakhawi et al. (2019) [8], the Berg Balance Scale and the Biodex Balance System were applied to assess functional balance and the steadiness of a circular force plate that was hanging and supported from above.

##### Protein Oxidation and Plasma Leptin Levels

In the study by Ordonez et al. (2010) [44], a Sport Tester PE3000 telemetric heart rate monitor and blood samples were used to measure carbonyl and protein content in the blood. In studies by Ordonez et al. (2013) [46], Ordonez et al. (2014) [48], and Rosety-Rodriguez et al. (2014) [51], a wireless heart rate monitor, bioelectrical impedance analysis, anthropometric tapes, and blood samples were applied to measure fat mass percentage and distribution, leptin and adipokine plasma levels, maximum oxygen uptake, waist circumference, and waist-to-hip ratio; the experiment by Rosety-Rodriguez et al. (2014) [51] additionally used plasma levels of IL-6 and high-sensitivity CRP; and the work by Ordonez et al. (2014) [48] additionally used fibrinogen and a1-antitrypsin.

##### Executive Function, Cognitive Performance and Verbal Fluency

In studies by Chen et al. (2015) [1], (2016) [2], and (2019) [3], protocols such as the Peabody Picture Vocabulary Test, Physical Activity Readiness Questionnaire, and vision and hearing tests were carried out. The time between the presence of a possible stimulus until the initiation of movement, the Knock-Tap test, and the Dimensional Change Card Sort Test were used in studies by Chen et al. (2015) [1] and (2016) [2] to assess executive function. Chen et al. (2016) [2] performed an experiment to evaluate cognitive performance, where participants were asked to respond to a blue light by pressing a specific button with their right index finger, and to a white light by pressing a different button with their left index finger. Chen et al. (2019) [3] conducted a study in which participants were asked to complete a verbal test. The initial 2 parts of the test required them to list as many words as they could think of that were associated with specific categories such as animals or food and drink, within a 60 s time limit. The final 2 parts of the test required them to list words beginning with the letters S and F, also within a 60 s time limit.

### 3.2. Outcomes

Two of the studies did not provide sufficient data for calculating mean differences (MD); therefore, they are only presented in narrative form. Ulrich et al. (1992) [31] tested alternating stepping patterns of 11-month-old infants with Down syndrome. The infants produced alternating steps on a moving treadmill, contrary to on a steady treadmill on which they produced no steps. There was no statistically significant difference in the number of steps taken at each of the three speeds of the treadmill. In the study it was found that the participants took a higher number of alternating steps (241 steps) than single (101), parallel (22), or double (4). In the study by Ulrich et al. (1995) [32], correlation was found between the development and shifting between stepping patterns produced by treadmill stimulation in infants with DS. The authors found that infants produce more alternating steps while growing up and a there is a shift from different step types to consistent and dominant alternation in stepping. The study conducted by Ulrich et al. (2001) [33] aimed to investigate whether infants with DS undergoing treadmill training achieved earlier development of three specific locomotor behaviors when compared to a control group. The mean advantages of those who participated in the treadmill training compared to the control group are that they were able to raise themselves to stand in 60 days, walk with help in 73 days, and walk independently after 101 days. However, in the comparison with the control group, only results referring to the walking with help reached statistical significance. Carmeli et al. (2002) [34] performed a study, the aim of which was to evaluate how treadmill exercise impacts leg strength and dynamic balance in older individuals diagnosed with DS. In the experimental group, peak torque %BW, and average power %BW of quadriceps and hamstrings of aged individuals with DS significantly increased after completing the treadmill protocol. Dynamic balance performance was also significantly improved in the walking group. The study by Carmeli et al. (2004) [35] focused on walking performance of elderly people with DS and intermittent claudication. Upon completion of the training program, all participants exhibited notable enhancements in their walking speed, distance, and duration. Individuals experiencing intermittent claudication also reported a reduction in pain levels. Additionally, significant improvements were observed in the participants’ blood hemodynamic parameters. Wu et al. (2007) [36] measured basic gait parameters and walking onset. There were three groups, which differed in walking experience (which was adjusted for in data analysis): control, low-, and high-intensity treadmill training. The high-intensity training group started walking at a younger age in comparison to the control. In terms of walking onset, there were no differences between low- and high-intensity training groups. An analysis conducted after the study showed that, aside from stride length, there were no significant differences in gait parameters between the groups. The high-intensity (HI) group had a significantly greater stride length compared to the control (C) group, while there was no significant difference between the low-intensity-generalized (LG) and HI groups. Stride length was the gait parameter that mainly contributed to the difference between the groups. The HI group showed a significantly longer stride length than the C group. Overall, the results suggest that the HI treadmill intervention led to earlier walking onset and advanced gait patterns, particularly in terms of stride length, in infants with DS. Wu et al. (2008) [37] measured the percentage of locomotor strategy and adjustment in obstacle clearance in a group of new walking toddlers with DS. The study participants trained in two different protocols: high and low intensity. The high-intensity group (84.3%) used walking strategies to avoid the obstacle more often than the low-intensity group (67.8%). There were no significant differences between the groups in terms of gait parameters in the five steps before approaching the obstacle. The study by Ulrich et al. (2008) [38] focused on stepping and motor development while comparing two groups: one with high-intensity and second with low-intensity treadmill training. There was no notable distinction between the groups in terms of the quantity of alternating steps taken at the start of the study. The group that engaged in high-intensity activities achieved all of the motor milestones more quickly than the group with low-intensity activities. However, only the accomplishment of item 52, which involved raising oneself to a standing position, was deemed statistically significant. The study by Angulo-Barroso et al. (2008) [39] compared physical activity in individuals with DS performing two treadmill training protocols: high intensity and low intensity, respectively. Infants receiving the high-intensity training had higher levels of High-act than infants in the low-intensity training group. Infants in the low-intensity group had higher duration of Low-act. The study by Angulo-Barroso et al. (2008) [40] explored the long-term effects of high-intensity and low-intensity treadmill training on gait development in walkers with DS. Six basic gait parameters were measured: normalized velocity, cadence, step length, step width, double support percentage, and dynamic base. On average, the high-intensity group performed better, producing higher normalized velocity and cadence, and lower double support percentage. Moreover, both groups significantly reduced foot rotation asymmetry over time, but no difference was observed between the groups. The study by Mendonca et al. (2009) [41] had a single group design, which measured the effect of treadmill training on submaximal and peak aerobic capacity. After the intervention, researchers observed decreased fat mass, increased absolute fat-free mass, and improved peak exercise capacity. There were no significant differences in participants’ body weight, BMI, resting VO2, or heart rate. In the study by Looper et al. (2010) [42], researchers compared the effect of treadmill training combined with orthoses (experimental group) and treadmill training alone (control group). In both groups, all scores in the Gross Motor Function Measure were increased, although the control group had higher scores in the standing and walking, running, and jumping subscales. In their research, Wu et al. (2010) [43] conducted a comparison between low-intensity and high-intensity treadmill training groups in terms of joint kinematics. Results showed that both groups made significant progress in terms of joint kinematics during gait follow-up. However, in the high-intensity training group, peak ankle plantar flexion occurred at or before toe-off, and there was an increase in the forward thigh swing after toe-off time. The study by Ordonez et al. (2012) [44] explored the correspondence with treadmill training and protein oxidation based on the carbonyl content in individuals with DS. In the intervention group, plasmatic carbonyl content significantly decreased, in contrast to the control group in which the researchers observed no significant changes. El-Meniawy et al. (2012) [45] compared the effects of suspension therapy and treadmill training on balance in children with DS. The mean differences in overall, anterior-posterior, and medio-lateral stability indexes suggest that both interventions are beneficial for balance; however, the Student *t*-test showed no significant differences between the groups. The study by Lin et al. (2012) [47] measured the strength of lower-extremity muscle and agility in two groups: the first experimental, which performed treadmill training, and the second, control group. Significant differences in muscle strength were observed for hip flexors, extensors and abductors, knee flexors and extensors, and ankle plantar flexors in the experimental group. Treadmill training also increased participants’ agility scores. The study by Ordonez et al. (2013) [46] explored the anti-inflammatory effect of treadmill training by measuring leptin levels in obese women with DS. Participants were divided into control and experimental groups. There were no significant changes in adiponectin plasma levels between the groups, but they differed in leptin plasmatic levels, which decreased in the training group. Fat mass percentage and waist-to-hip ratio were also reduced in the training group. Moreover, researchers found a positive association between leptin and WHR and a negative association between adiponectin and waist circumference. The study by Ordonez et al. (2014) [48] assessed the impact of treadmill training on plasmatic levels of pro-inflammatory cytokines and body composition in obese women with DS. In the intervention group, plasmatic levels of TNF- α, IL-6, high-sensitivity C-reactive protein, and fibrinogen were significantly decreased. Similarly, both waist-to-hip ratio and fat mass percentage decreased in the training group. No significant changes were observed in the control group. The experiment by Rodenbusch et al. (2013) [49] was a single group design study that aimed to explore the effect of treadmill inclination on gait of children with DS. In spatio-temporal variables, a reduction in cadence and an increase in cycle and swing time were observed, after the upward treadmill inclination was increased. In angular variables, researchers observed an increased angle at initial contact in hip, knee, and ankle joints, increased maximum flexion angle in the hip joint, maximum plantarflexion at pre-swing, and an increase in maximum dorsiflexion in stance, while walking on an inclined surface. The study by Chen et al. (2014) [50] explored the effect of treadmill training on grip strength in individuals with DS. Both study groups’ post-test scores were significantly different, although the intervention group had higher grip strength scores. In the study by Rosety-Rodriguez et al. (2014) [51], researchers analyzed the longitudinal effect of treadmill training on pro-inflammatory cytokines and body mass composition. There were no significant changes over time in the control group. The intervention group showed a significant increase in plasma levels of IL-6 and hs-CRP (P = 0.026) at 3 months following the completion of the training program, compared to the post-test results. Moreover, there was a significant increase in both IL-6 and hs-CRP at 6 months after training, compared to the measurements taken just 1 month after the end of the intervention program. The study by Chen et al. (2015) [52] focused on the effect of treadmill training on choice-response time, attention shifting, and inhibition. The ANCOVA showed that both choice-response time and attention shifting did not significantly differ between the study groups. A statistically significant difference was present only in inhibition favoring the exercise group. Chen et al. (2016) [53] explored the dose–response relationship between exercise and cognitive performance represented by information processing, attentional switching, and inhibitory control. The ANCOVA and polynomial contrast showed no significant changes nor any trend in terms of attentional shifting. Inhibitory control improved linearly with intensity of training. Regarding information processing speed, the low-intensity group performed better than both the control group and the high-intensity group. The purpose of the study by Chen et al. (2019) [54] was to assess the relationship between exercise and cognitive functions, and especially their verbal fluency aspects. The ANCOVA, Levene’s test, and normality check showed only a significant quadratic trend for semantic fluency. The study by Alsakhawi et al. (2019) [8] compared treadmill training and core stability exercises to the effects of treatment in the control group (receiving standard physiotherapy) in order to determine which intervention is more effective in improving balance in children with DS. Functional balance and the overall stability index were significantly higher in stability and treadmill training groups MD, respectively, although there was no significant difference between intervention groups.

Overall, the studies suggest that treadmill training can have positive effects on various aspects of motor development, such as increasing the number of alternating steps, improving leg strength and dynamic balance, and enhancing walking performance in individuals with DS. Additionally, treadmill training has been found to have anti-inflammatory effects and improve body composition in obese individuals with DS. The studies also suggest that treadmill training can improve cognitive functions, particularly inhibitory control and information processing speed, in individuals with DS.

### 3.3. Ethical Issues Concerning Studies Involving Infants and Children

In the analysis of ethical approvals and informed consent across the studies involving infants and children, there is a broad yet uneven adherence to the established ethical guidelines of biomedical research. Studies involving infants, including those by Ulrich et al. (1992) [31], Ulrich et al. (1995) [32], Ulrich et al. (2001) [33], Wu et al. (2007) [36], Wu et al. (2008) [37], Ulrich et al. (2008) [38], Angulo-Barroso et al. (2008) [40], Looper et al. (2010) [42], and Wu et al. (2010) [43], demonstrate a general compliance with the principle of informed consent. They reported obtaining consent from parents or legal guardians, thereby respecting their autonomy and their right to decide on their infants’ participation in their respective studies. However, the reporting on the acquisition of ethical approval from relevant institutional review boards or ethics committees in these studies was inconsistent. The studies by Ulrich et al. (2001) [33], Wu et al. (2007) [36], Wu et al. (2008) [37], Ulrich et al. (2008) [38], Looper et al. (2010) [42], Angulo-Barroso et al. (2008) [40], and Wu et al. (2010) [43] explicitly stated that they received ethical approval, indicating their commitment to the principles of beneficence and non-maleficence. These principles require researchers to ensure that the potential benefits of their research outweigh any potential harm to the participants. On the other hand, the studies by Ulrich et al. (1992) [31] and Ulrich et al. (1995) [32], while acknowledging the acquisition of informed consent, did not explicitly mention obtaining ethical approval.

When it comes to studies involving children, such as those by Ordonez et al. (2012) [44], El-Meniawy et al. (2012) [45], Lin et al. (2012) [47], Rodenbusch et al. (2013) [49], and Chen et al. (2019) [54], the adherence to the ethical principles of informed consent and ethical approval appears to be more consistent. All these studies reported obtaining informed consent from parents or legal guardians, respecting their autonomy and their right to decide on their children’s participation in the research. Furthermore, the studies by Ordonez et al. (2012) [44], Rodenbusch et al. (2013) [49], and Chen et al. (2019) [54] explicitly stated that they received ethical approval from the respective institutional ethics committees or review boards. These reports confirm their commitment to ensuring the safety and well-being of the child participants in their research. However, the studies by El-Meniawy et al. (2012) [45] and Lin et al. (2012) [47] reported obtaining informed consent but did not specify the acquisition of ethical approval.

In conclusion, while most studies demonstrate a broad adherence to the principles of informed consent and ethical approval, there is a need for more consistent and explicit reporting of ethical aspects in research focusing on infants and children with DS, particularly regarding ethical approval. This is crucial not only to uphold the ethical integrity of research, but also to ensure the trust of the public and the scientific community in the research process and its outcomes.

### 3.4. Risk of Bias 

In this section we present the results of risk of bias assessment for 15 studies: 9 randomized controlled trials and 6 parallel randomized trials with no control group. A summary of the analysis across all domains for each individual study can be seen in Table 2 and the proportion of studies in each domain is shown below. Only three studies met the criteria for an overall low risk of bias, six studies were identified as having some concerns, and six were identified as having a high risk of bias.

- Domain 1: Risk of bias arising from randomization process

Six studies had a well-described randomization process [8,36,40,43,47,48]; therefore, we identified them as having low risk of bias in this domain. We assessed seven studies [8,31,37,38,39,46,52] as having some concerns, because of evasive descriptions of randomization and concealment and lack of differences between study groups. Two studies had high risk of bias in this domain due to no allocation concealment and baseline differences between study groups [42,50].

- Domain 2: Risk of bias due to deviations from the intended interventions

Seven studies had no dropouts and no deviations from the outcome; on that account we judged them as having low risk of bias [8,33,37,45,46,47,48]. Seven studies were marked as having some concerns because of a lack of intent to treat analysis when dropouts occurred. [36,38,39,40,43,50,52]. The study by Looper et al. (2010) had a high risk of bias in this domain since the lack of concealment of caregivers led to deviations from the protocol [42].

- Domain 3: Risk of bias due to missing outcome data

Nine studies were assessed as having low risk of bias because there were no missing data [1,2,6,7,8,9,11,12,13]. Five studies had a high risk of bias because of missing data and “per protocol” or “as treated” analysis model; also, no sensitivity analysis was conducted [5,6,9,10,14]. In the study by Wu et al. (2010) [12], the participants also dropped out during the trial, but the recruited group was 10% bigger as a precaution of missing data.

- Domain 4: Risk of bias in measurement of the outcome

We found no potential misconduct in this domain; in most studies, researchers used adequate scales and tools for an outcome measure. Furthermore, in most cases the outcome assessor was unaware of the group assignment or this knowledge was unlikely to affect the assessment. Therefore, none of the included studies had a high risk of bias in this domain and only one was judged as having some concerns, because of the authors’ statement that the assessor was not blinded and this is likely to interfere with the results [12].

- Domain 5: Risk of bias in selection of the reported results

All of the included reports had a low risk of bias in domain 5, because we found no evidence that authors used multiple outcome measurements or analysis.

**Table 2:** Risk of bias: authors’ judgment about risk of bias for each domain for each included study eligible for assessment. Colors used in the table represent—green: low risk of bias; yellow: some concerns about bias; red: high risk of bias.

**Randomized Controlled Trials**	**Domain 1**	**Domain 2**	**Domain 3**	**Domain 4**	**Domain 5**	**Overall**
Ulrich et al. (2001) [33]	Some concerns	Low risk	Low risk	Low risk	Low risk	Some concerns
Wu et al. (2007) [36]	Low risk	Some concerns	High risk	Low risk	Low risk	High risk
Looper et al. (2010) [42]	HIgh risk	High risk	High risk	Some concerns	Low risk	High risk
Lin et al. (2012) [47]	Low risk	Low risk	Low risk	Low risk	Low risk	Low risk
Ordonez et al. (2013) [46]	Some concerns	Low risk	Low risk	Low risk	Low risk	Some concerns
Ordonez et al. (2014) [48]	Low risk	Low risk	Low risk	Low risk	Low risk	Low risk
Chen et al. (2014) [50]	HIgh risk	Some concerns	Low risk	Low risk	Low risk	High risk
Chen et al. (2015) [52]	Some concerns	Some concerns	High risk	Low risk	Low risk	High risk
Alsakhawi et al. (2019) [8]	Low risk	Low risk	Low risk	Low risk	Low risk	Low risk
**Randomized Trials with no Control**	**Domiain 1**	**Domain 2**	**Domain 3**	**Domain 4**	**Domain 5**	**Overall**
Wu et al. (2008) [37]	Some concerns	Low risk	Low risk	Low risk	Low risk	Some concerns
Ulrich et al. (2008) [38]	Some concerns	Some concerns	High risk	Low risk	Low risk	High risk
Angulo-Barroso et al. (2008) [39]	Some concerns	Some concerns	High risk	Low risk	Low risk	High risk
Angulo-Barroso, Wu et al. (2008) [40]	Low risk	Some concerns	Low risk	Low risk	Low risk	Some concerns
Wu et al. (2010) [43]	Low risk	Some concerns	Some concerns	Low risk	Low risk	Some concerns
EL-Meniawy et al. (2012) [45]	Some concerns	Low risk	Low risk	Low risk	Low risk	Some concerns

## 4. Discussion

Down syndrome is a genetic disorder impacting physical and cognitive development. Exercise, particularly treadmill training, offers benefits such as enhanced strength, balance, and physical functioning. This review examined treadmill training’s advantages for individuals with DS by evaluating 25 studies with 687 participants, assessing its effectiveness across all ages [8,31,32,33,34,35,36,37,38,39,40,41,42,43,44,45,46,47,48,49,50,51,52,53,54]. Because we were unable to conduct a meta-analysis, we narratively reported results. Unlike other articles, we considered all benefits of treadmill use for individuals with Down syndrome across various domains [21,22,23,24,25,26,27]. Compared to other physical activities such as aerobic exercise, swimming, and cycling, treadmill training has proven to be one of the more effective, simpler, and safer methods of helping people with DS with their various health problems [55,56,57]. The review of the studies faced several challenges. One of them was a wide spectrum of age groups in the examined populations. The manuscripts we analyzed referred to infants [31,32,33,36,37,38,39,40,42,43], children and adolescents [44,45,47,49,54], and adult DS patients [34,35,41,46,48,50,51,52,53,54]. The number of participants in the study group also varied among the papers, with two cases having very small sample sizes of less than ten [31,32]. Most of the studies had an intermediate size, between 10 and 30 participants [34,35,41,46,48,50,51,52,53,54]. Only three papers had larger study groups with more than thirty participants [8,44,47]. All the studies applied treadmill training as the form of intervention; however, there were some differences concerning speed, duration, and inclination of the treadmill exercise. In the studies involving infants [31,32,33,42], the participants were supported by other individuals, trainings were repeated up to five times a week, and the whole program lasted from eight repetitions of training to a longer period of several months. In the studies involving adults [34,35,36,37,40,41,43,44,45,46,47,48,49,51], the trainings lasted from 6 to 40 min and were repeated from 3 to 5 times a week. The training program lasted from 3 months to 14 weeks. Various assessment tools were used to evaluate outcomes such as motor skills, cardiovascular function, cognitive performance, and verbal fluency. These tools included scales for infant development, camera recordings, balance and motion capture systems, heart rate monitors, and tests for cognitive and verbal abilities. Effects measures in mean differences (MDs) of the analyzed studies could be presented for eighteen studies, and two of the studies did not provide sufficient data for calculating MD. The most commonly observed outcomes of the trainings were significant improvements in walking speed, distance, and duration, and better motor development in children.

This systematic review examining the benefits of treadmill training for individuals with Down syndrome (DS) unveils several important implications for practice. Firstly, treadmill training can accelerate walking onset and enhance gait development in children with DS, helping them walk with improved patterns at an earlier age. Secondly, adults with DS can experience anti-inflammatory advantages and cognitive function improvements through treadmill training.

As a form of exercise that is both safe and easy to perform for people of all ages, treadmill training presents a practical, accessible solution for those with DS. In essence, these findings emphasize the significance of treadmill training as a safe, effective method for boosting physical and cognitive functions in people with DS.

To provide a more comprehensive understanding of the effects of treadmill training, future studies should concentrate on several key areas: assessing cognitive function, including memory and attention; determining the optimal duration and frequency of treadmill training for maximum benefits; investigating the potential of treadmill training as a preventative measure against Alzheimer’s disease in people with DS; and employing an appropriate control group, such as individuals with DS who do not partake in treadmill training, to accurately gauge the intervention’s effects.

Despite the comprehensive approach and extensive analysis, this manuscript has several limitations that should be acknowledged. Firstly, the studies included in this review were conducted at different times and locations, which could have resulted in variations in methodology and results. Secondly, the majority of studies included were conducted on small sample sizes, which may limit the generalizability of the findings. Thirdly, the studies varied in terms of the age ranges of participants, which may have impacted the effectiveness of the interventions. Fourthly, the studies also varied in terms of the intervention duration and frequency, which makes it difficult to draw conclusions about the optimal parameters for effective intervention. Lastly, the risk of bias assessment revealed that some studies had high risk of bias, particularly in the domains of randomization and missing outcome data, which may have influenced the results. These limitations suggest a need for further well-designed studies with larger sample sizes and consistent intervention protocols to fully explore the effects of treadmill training on individuals with Down syndrome.

## 5. Conclusions

Our systematic review found that treadmill training can help children with Down syndrome develop walking and gait patterns at an earlier age, and can provide anti-inflammatory and cognitive benefits for adults with DS. Treadmill training is safe and easy to implement, and has the potential to improve physical and cognitive functions. Future studies should focus on assessing cognitive function, determining the optimal duration and frequency of training, exploring the potential of treadmill training as a preventative intervention for Alzheimer’s disease, and using appropriate control groups.

## Figures and Tables

**Table 1:** Study characteristics and participants. Abbreviations: SG—study group, CG—control group, EG—experimental group, LG—lower-intensity-generalized training group, HI—higher-intensity-individualized training group, LG—generalized low intensive training, MI—moderate-intensity exercise.

No.	Study	Duration	Design	Intervention	Age	Co-Morbidities
1	Ulrich et al. (1992) [31]	11 months	Single group design	Supported treadmill stepping for infants with DS	7 months	Congenital heart defects (n = 5)
2	Ulrich et al. (1995) [32]	4–21 months	Single group design	Longitudinal supported treadmill stepping for infants with DS	8–11 months	Congenital heart defects (n = 5)
3	Ulrich et al. (2001) [33]	Until independent walking	Randomized controlled trial	Treadmill stepping practice for infants with DS	307.4 days	Congenital heart disease requiring surgery (SG: n = 7, CG: n = 2)
4	Carmeli et al. (2002) [34]	6 months	Parallel group design	Treadmill walking program for adults with DS	63 years	SG: Cardiac disease (n = 2)
5	Carmeli et al. (2004) [35]	15 weeks	Parallel group design	Treadmill walking program for ID adults with arterial occlusive disease	SG: 65.5 years, CG: 62 years	Arterial occlusive disease
6	Wu et al. (2007) [36]	Until 3 independent steps + 1 and 3-month follow-up	Randomized controlled trial	Different treadmill interventions for infants with DS	SG: LG—21.4 months, HI—19.2 months, CG: 23.9 months	Not reported
7	Wu et al. (2008 [37])	LG group—11 months, HI group—9.6 months + 1-year follow-up	Randomized trial (no control)	Treadmill interventions for newly walking toddlers with DS	HI group: 9.65 months, LG group: 10.40 months	Not reported
8	Ulrich et al. (2008) [38]	Until 3 independent steps	Randomized trial (no control)	Individualized, progressively intense treadmill training for infants with DS	HI group: 9.65 months, LG group: 10.40 months	Congenital heart defects (HI: n = 8, LG: n = 6)
9	Angulo-Barroso et al. (2008) [39]	15 months + 1-year follow-up	Randomized trial (no control)	Higher intensity, individualized TMT protocol for infants with DS	HI group: 9.65 months, LG group: 10.40 months	Not reported
10	Angulo-Barroso, Wu et al. (2008) [40]	15 months + 1-year follow-up	Randomized trial (no control)	Long-term effect of different treadmill interventions on gait patterns in infants with DS	HI group: 9.7 months, LG group: 10.40 months	Not reported
11	Mendonca et al. (2009) [41]	12 + 28 weeks	Single group design	28-week training program for DS males to improve aerobic capacity and locomotor economy	34.5 years	Not reported
12	Looper et al. (2010) [42]	Until 3 independent steps + 1-month follow-up	Randomized controlled trial	Early orthosis use combined with treadmill training in infants with DS vs. treadmill training alone	SG: 578 days, CG: 642 days	Not reported
13	Wu et al. (2010) [43]	Until 3 independent steps + 1-year gait follow-up	Randomized trial (no control)	Different treadmill interventions on joint kinematic patterns in infants with DS	LG: 35.7 months, HI: 75 months	Not reported
14	Ordonez et al. (2010) [44]	12-week training	Parallel group design with matched control	Aerobic training for reducing protein oxidation	16.3 years	Not reported
15	El- Meniawy et al. (2012) [45]	3 months intervention + 3-month follow-up	Randomized trial (no control)	Treadmill training vs. suspension therapy on balance in children with DS	9.34 years	Not reported
16	Ordonez et al. (2013) [46]	10-week aerobic training program	Randomized controlled trial	Aerobic training on plasma adipokines in obese women with DS	EG: 24.7 years, CG: 25.1 years	Obesity, mild ID
17	Lin et al. (2012) [47]	6-week program + 6-week follow-up	Randomized controlled trial	Strength and agility training for adolescents with DS	SG: 10.6 years, CG: 11.2 years	Not reported
18	Ordonez et al. (2014) [48]	10-week aerobic training program	Randomized controlled trial	Aerobic training on pro-inflammatory cytokines and acute phase proteins in women with DS	EG: 24.7 years, CG: 25.1 years	Obesity
19	Rodenbusch et al. (2013) [49]	Only intervention	Single group design	Effects of upward treadmill inclination on gait of children with DS	8.43 years	Not reported
20	Chen et al. (2014) [50]	~20 min (only intervention)	Randomized controlled trial	Relation between grip strength, anthropometric factors, and aerobic exercise impact on grip strength in young men with DS	EG: 21.76 years, CG: 17.77 years	Not reporte
21	Rosety-Rodriguez et al. (2014) [51]	10-week aerobic training program + 6-month follow-up	Parallel group design with matched control	Reduced inflammation effects maintenance after aerobic program completion	EG: 24.7 years, CG: 25.1 years	Obesity, mild ID
22	Chen et al. (2015) [52]	~20 min (only intervention)	Randomized controlled trial	Impact of single exercise intervention on executive function in young adults with DS	EG: 23.45 years, CG: 20.58 years	Not reported
23	Chen et al. (2016) [53]	~20 min (only intervention)	Parallel group design with matched control	Dose-response relationship between acute exercise intensity and cognitive performance	MI: 23.7 years, HI: 22.10 years, CG: 19.11 years	Not reported
24	Chen et al. (2019) [54]	~20 min intervention + 5–10 min rest + ~1.5 h verbal tests	Parallel group design with matched control	Relationship between acute exercise and verbal fluency	MI: 21.42 years, HI: 22.70 years, CG: 20.58 years	Not reported
25	Alsakhawi et al. (2019) [8]	4 months	Randomized controlled trial	Core stability training vs. treadmill exercises on balance in children with DS	4.59 years	Not reported

## Data Availability

No new data were created.
